# Supplementary material for: The interactome of intact mitochondria by cross-linking mass spectrometry provides evidence for coexisting respiratory supercomplexes
Source: Mol Cell Proteomics. 2017 Dec 8;17(2):216–32. doi: 10.1074/mcp.RA117.000470 (PMC5795388; doi:10.1074/mcp.RA117.000470)
Supplement: Supplemental Data [file supp_RA117.000470_133922_0_supp_23978_fzffwf.zip › spectra_annotation/mito_spectra_annotation/361-1-6-1-9-1.pdf]

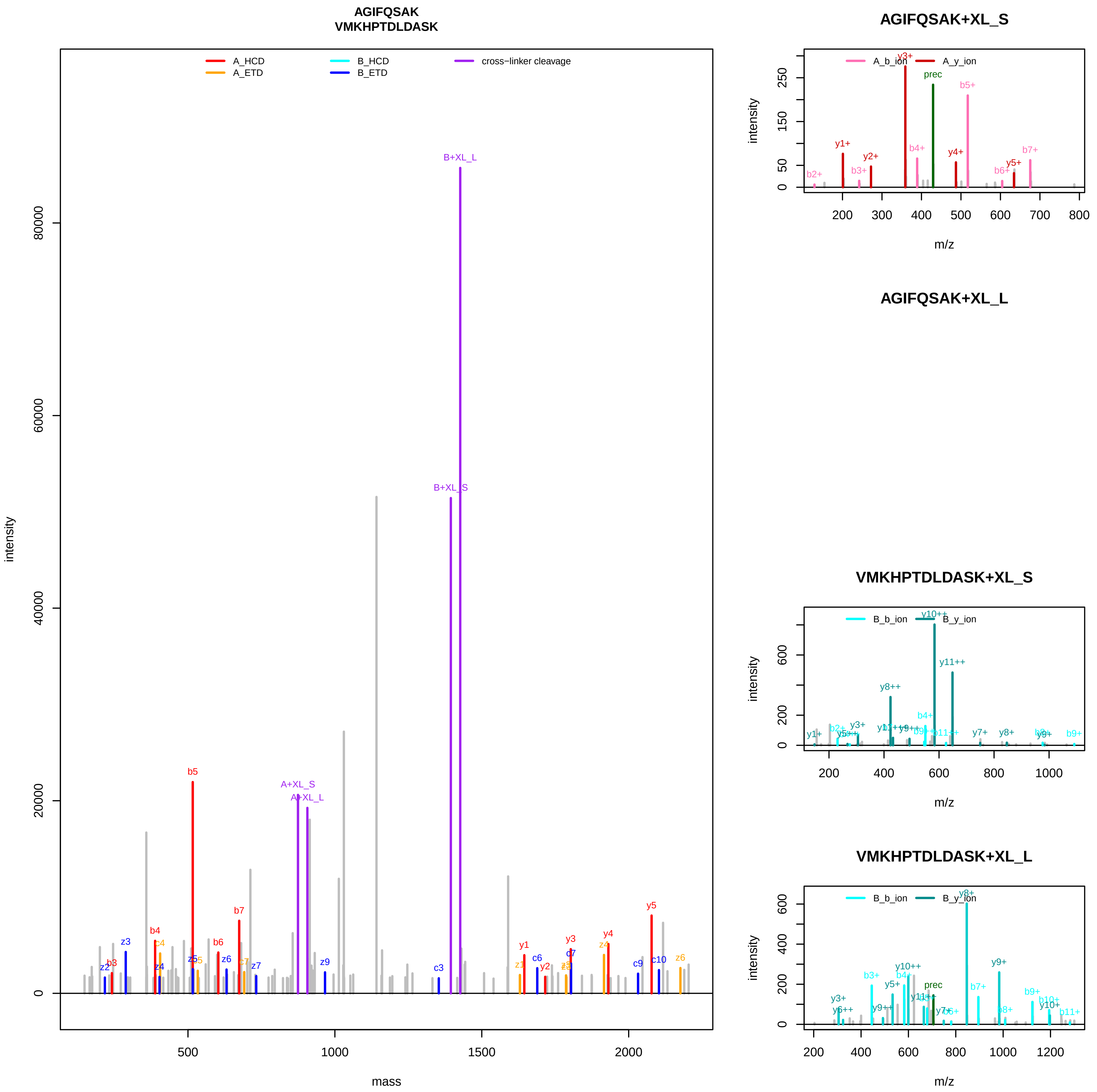
AGIFQSAK
VMKHPTDLDASK
A_HCD
A_ETD
B_HCD
B_ETD
cross−linker cleavage
intensity
mass
B+XL_L
B+XL_S
A+XL_S
A+XL_L
b3
b4
b5
b6
b7
c4
c5
c7
z2
z3
z4
z5
z6
z7
z9
c3
c6
c7
c9
c10
y1
y2
y3
y4
y5
z1
z3
z4
z6
0
20000
40000
60000
80000
500
1000
1500
2000
AGIFQSAK+XL_S
A_b_ion
A_y_ion
prec
b2+
b3+
b4+
b5+
b6+
b7+
y1+
y2+
y3+
y4+
y5+
m/z
AGIFQSAK+XL_L
VMKHPTDLDASK+XL_S
B_b_ion
B_y_ion
y10++
y11++
y8++
b4+
y1+
b2+
y3+
y7+
y8+
b9+
VMKHPTDLDASK+XL_L
y8+
y9+
b3+
y5+
b4+
y10++
prec
b7+
b9+
b10+
y10+
b11+
y3+
y6++
y9++
y11++
y7+
